# Comparison of the Effect of High-Intensity Laser Therapy (HILT) on Skin Surface Temperature and Vein Diameter in Pigmented and Non-Pigmented Skin in Healthy Racehorses

**DOI:** 10.3390/ani11071965

**Published:** 2021-06-30

**Authors:** Paulina Zielińska, Maria Soroko, Kevin Howell, Maria Godlewska, Weronika Hildebrand, Krzysztof Dudek

**Affiliations:** 1 Department of Surgery, Faculty of Veterinary Medicine, Wroclaw University of Environmental and Life Sciences, Plac Grunwaldzki 51, 50-366 Wroclaw, Poland; godlewskamaria23@gmail.com (M.G.); weronika.hildebrand@gmail.com (W.H.); 2 Institute of Animal Breeding, Wroclaw University of Environmental and Life Sciences, Chelmonskiego 38C, 51-630 Wroclaw, Poland; maria.soroko@upwr.edu.pl; 3 Microvascular Diagnostics, Institute of Immunity and Transplantation, Royal Free Hospital, Pond Street, London NW3 2QG, UK; k.howell@ucl.ac.uk; 4 Faculty of Mechanical Engineering, Wroclaw University of Technology, Lukasiewicza 7/9, 50-231 Wroclaw, Poland; krzysztof.dudek@pwr.edu.pl

**Keywords:** high-intensity laser therapy, fetlock joint, physiotherapy, thermography, skin surface temperature

## Abstract

**Simple Summary:**

High-intensity laser therapy (HILT) is used in the treatment of horses, but little is known about the differences in the impact of HILT performed on pigmented and non-pigmented skin. The aim of this study was to assess differences in the influence of HILT on skin surface temperature and vein diameter in a group of healthy racehorses with pigmented and non-pigmented skin in the treatment area. The hypothesis was that HILT would cause a greater increase in skin surface temperature and vein diameter in horses with pigmented skin compared to non-pigmented skin. Ten Thoroughbreds with pigmented skin and ten Thoroughbreds with non-pigmented skin in the treatment area received HILT. Changes in the vein diameter and skin surface temperature of the irradiated area were measured before and after HILT. The HILT treatment caused an increase in the pigmented skin surface temperature and a decrease in the non-pigmented skin surface temperature, while the vein diameter increased in both groups. In conclusion, melanin content in the epidermis plays an important role in light energy absorption and photothermal effects. Determining the physiological and clinical effects of HILT performed on pigmented and non-pigmented skin will help clinicians choose appropriate HILT parameters.

**Abstract:**

The aim of the study was to assess differences in the influence of high-intensity laser therapy (HILT) on the skin surface temperature and vein diameter of the lateral fetlock joint region in a group of racehorses with pigmented and non-pigmented skin in the treatment area. Twenty Thoroughbreds were divided into two equal groups: pigmented and non-pigmented skin groups. Each horse received the same HILT treatment. Just before and immediately after HILT, thermographic examination was performed to measure the skin surface temperature and ultrasonographic examination assessed the lateral digital palmar vein diameter. After HILT, the pigmented skin surface temperature increased, while the non-pigmented skin surface temperature decreased, and the difference between both groups was significant (*p* < 0.001). The vein diameter increased after HILT in horses with pigmented and non-pigmented skin, but the difference between both groups was not significant (*p* = 0.14). In conclusion, melanin content in the epidermis plays an important role in light energy absorption and photothermal effects. The vein diameter changes after HILT application indicated that the increase in vessel diameter may partly depend on photothermal mechanisms occurring in irradiated tissue. Further research is necessary to describe the physiological and clinical effects of HILT performed on pigmented and non-pigmented skin.

## 1. Introduction

Light amplification by stimulated emission of radiation (LASER) describes coherent beams of single wavelengths in the ultraviolet–visible to infrared spectrum that can be emitted in a continuous wave or pulsed mode [1]. Laser therapy with class IIIb lasers with an average power not exceeding 500 mW (classified by the Center for Devices and Radiological Health (CDRH)) is called low-level laser therapy (LLLT). Therapeutic laser devices with a much higher power (classified by CDRH as class IV lasers) are used in high-intensity laser therapy (HILT). The advantage of HILT over LLLT is that HILT is able to reach and stimulate larger and deeper joints and areas that are difficult to reach with LLLT. In addition, during a HILT session, a significantly greater amount of energy may be transferred into the tissue compared to LLLT [2,3].

In human medicine, HILT is successfully used in the rehabilitation of many orthopedic injuries and disorders [4,5,6]. This relatively novel treatment has been recently introduced in equine veterinary medicine. Only a few studies have evaluated the impact of HILT in the treatment of orthopaedic disorders in horses and are mainly focused on the management of tendon injuries. Promising results have been presented in our previous randomized controlled non-blind study, which focused on 26 horses with 29 cases of superficial digital flexor tendon (SDFT), deep digital flexor tendon (DDFT) and suspensory ligament (SL) injuries; the horses were assigned to either a group treated with HILT or a non-treated (control) group. In that study, improvement was found in the pain, swelling and lameness assessed after treatment in the group treated with HILT compared with the control group. Moreover, in ultrasound examination for lesion percentage reduction, there was a statistically significant improvement in the group treated with HILT in comparison with the control group [7]. Similar clinical results using HILT were described by Pluim et al. [8], who treated tendinopathy and desmopathy with HILT or with HILT and other therapies on a group of 150 sport horses, but the study did not include a control group. Other studies have indicated the effectiveness of HILT in the treatment of bone spavin [9] and medial carpal collateral ligament injury [10]. However, both studies described single clinical cases and mainly focused on reduction of clinical symptoms of the disease (such as pain, lameness and swelling) after HILT.

Therefore, it is important to study the reaction of healthy horse tissue to HILT and further investigate the technique’s ability to activate and modulate the physiological response during the healing process.

One of the preconditions for improving HILT procedures in equine veterinary medicine is the determination of photothermal effects, which are a result of the transformation of absorbed light energy to heat [11]. It has been demonstrated that thermal effects reduce muscle spasm, increase tissue perfusion and relieve pain [12,13,14]. The components of the tissue that absorb the photons are chromophores, such as melanin, hemoglobin and water [11]. Studies on humans have confirmed that dark, strongly melanized (pigmented) skin absorbs much more laser light energy than fair (non-pigmented) skin [15,16]. Large numbers of melanocytes located in the bottom layer of the epidermis can cause nonspecific light energy absorption and possible thermal injury [17]. Anderson et al. [18] showed that pigmented melanosomes are a major site of cutaneous damage caused by laser pulses in the visible and near-infrared light spectrum. Additionally, light absorption by melanin reduces the total amount of energy that should be reaching deeper tissues to give therapeutic effects. Effective laser therapy of pigmented skin can be achieved since the absorption coefficient of melanin decreases exponentially as the laser wavelength increase. Laser–tissue interaction mechanisms are a function of wavelength, power, and pulse duration. The penetration depth of the laser light depends on the optical properties of the tissue at the selected wavelength [19,20]. Therefore, HILT parameters must be carefully considered when the therapy is performed in treatment areas with dark skin [17,21].

Previous studies have indicated that HILT increases blood flow in soft tissue, which creates a favourable environment for biological repair and regeneration [22,23,24] and promotes analgesic effects [25]. The increase in blood flow depends on the photochemical and photothermic effects of high-energy laser light absorption [26], but the mechanisms and pathways through which HILT impacts vasodilatation and blood flow are still not completely understood [27]. In a previous study of ours, which involved racing Thoroughbreds being subjected to ultrasonographic and thermographic examination of the tarsal joint before and after HILT application, we observed a significant increment in the diameter of the cranial branch of the medial saphenous vein and an increase in surface temperature after HILT treatment [28]. However, the study did not control for skin pigmentation in the treatment area.

The present study was conducted to evaluate the influence of HILT on skin surface temperature and vein diameter with a comparison of the results on pigmented and non-pigmented skin at the lateral surface of the fetlock joint in clinically healthy racehorses. It was hypothesized that HILT treatment would lead to a greater increase in skin surface temperature and vein diameter in horses with pigmented skin in the treatment area in comparison to horses with non-pigmented skin. It was also hypothesized that a positive correlation between an increase in skin surface temperature and vein diameter would be found in both groups of horses. The fetlock joint was chosen as the anatomical measurement area to allow for the easy examination of superficial vein diameter.

## 2. Materials and Methods

### 2.1. Animals and Study Design

The study was conducted on twenty clinically healthy Thoroughbreds aged 3-4 years. Horses were in regular racing training at Partynice Race Course in Wroclaw (Poland) and were housed in individual boxes with common management and training regimes. The study was approved by the Local Ethical Committee for Experiments on Animals, Wroclaw, Poland (no 003/2020).

Horses were divided into two groups: a pigmented skin group (group A, n = 10) and a non-pigmented skin group (group B, n = 10). Horses from group A had pigmented skin in the distal part of the limb and treatment area (black hair), and horses from group B had non-pigmented skin in the distal part of the limb and treatment area (white hair) [29,30]. The HILT treatment area was localized to the lateral aspect of the fetlock joint and lateral palmar digital vein of the left or right hindlimb. The coat in the treatment area was shaved (blade size 0.8 mm). The area of shaved skin was 26 cm^2^ and was consistent for all horses. Each horse underwent thermographic examination directly after shaving to determine skin surface temperature at the lateral surface of the fetlock joint (skin surface temperature), followed by ultrasonographic examination to measure the diameter of the lateral palmar digital vein (vein diameter). The treatment area was then dried of ultrasound coupling gel with a paper towel and HILT was performed. Immediately after HILT treatment, the thermographic and ultrasound examination was repeated to assess changes in skin surface temperature and vein diameter.

### 2.2. Thermography

**Figure 1:**
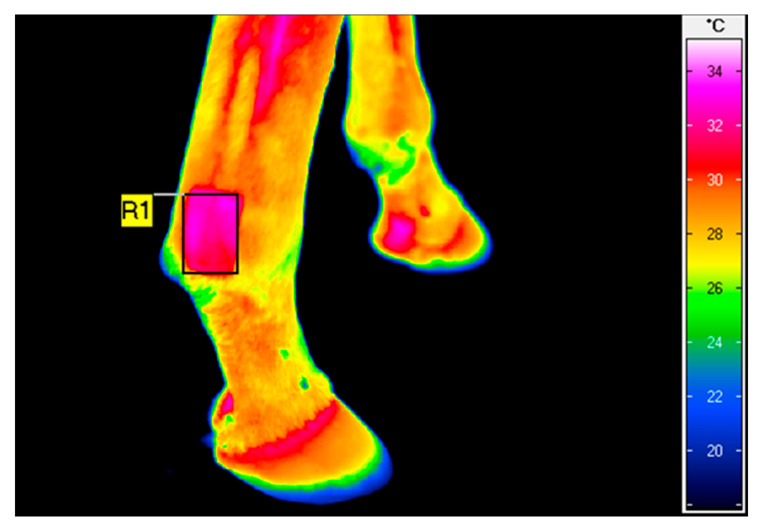
Example of thermographic image of the lateral aspect of the right non-pigmented hindlimb fetlock joint taken after HILT. The rectangular area (R1) indicates the average surface temperature of 31.7 °C.

Thermographic examination was performed using a Vario Cam hr resolution infrared camera (InfraTec, Dresden, Germany). The protocol for thermography was the same as that in our previous studies [28,31]. To minimize the effect of environmental factors, thermography was performed in an enclosed stable, outside of the horse box at an ambient temperature of approximately 10 °C. The thermographic images were all taken by the same operator. The distance between the animal and the camera was fixed for all imaging at 0.5 m, and the emissivity (ε) was set to 1 for all readings. The average surface temperature of a rectangular area placed over the shaved area (Figure 1) was calculated using IRBIS 3 Professional software (InfraTec, Dresden, Germany).

### 2.3. Ultrasound Examination

**Figure 2:**
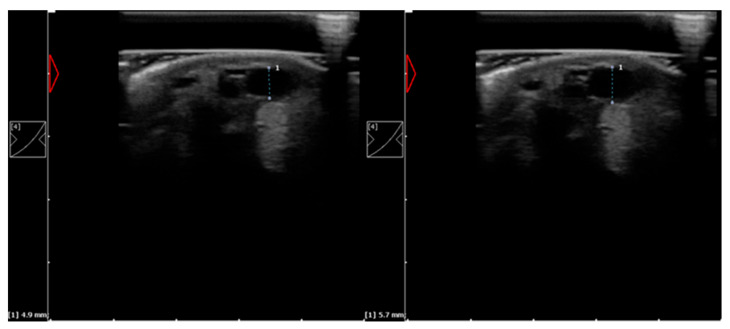
Example of ultrasound images of the lateral palmar digital vein with diameter measurement of the left pigmented hindlimb fetlock joint taken before (on the **left**) and after HILT (on the **right**).

Ultrasound examination was performed with a 10 MHz linear transducer (Dramiński^®^, Olsztyn, Poland). To achieve better image quality, an ultrasound probe standoff pad was used. Ultrasound scanning was conducted on weight-bearing limbs, and horses were restrained without sedation. All ultrasound examinations were performed by the same operator. During ultrasound examination, the examiner applied minimal pressure on the tissue to prevent changes in the vein diameter. A minimal amount of coupling gel was applied to minimize skin surface temperature changes. The lateral palmar digital vein was visualized in cross-sectional view, and the diameter of the vessel was measured (Figure 2).

### 2.4. High-Intensity Laser Therapy

Infrared laser light was generated by the class 4 laser Polaris HP S (Astar^®^, Bielsko-Biała, Poland) with an output power of 8 W (AlGaAs diode laser; wavelength: 808 nm; probe size: 5 cm^2^). The optical power was calibrated by an external service company. The irradiation was applied over the treatment area using a contact technique and manual scan with a handpiece positioned perpendicular to the tissue. In all horses, HILT was performed on a weight-bearing limb by the same person. All participants wore safety glasses (Astar^®^, Bielsko-Biała, Poland) to prevent retina injury from the laser beam. The treatment area was equal in size to the shaved area (26 cm^2^), and the duration of therapy was 203 s. The dose and parameters were chosen based on our previous study [28]: energy density: 25 J/cm^2^; power output: 4 W; frequency: 2000 Hz; and total energy dose: 650 J.

### 2.5. Statistical Analysis

Data were statistically analyzed using STATISICA v.13 (TIBCO Software Inc., Palo Alto, Santa Clara, CA, USA). For all measurable parameters, median (Me), lower quartiles (Q_1_), upper quartiles (Q_3_) and minimal and maximal values (Min–Max) were calculated. The distributions of values of skin surface temperature and vein diameter were verified as non-normal by the Shapiro–Wilk test. The independent non-parametric Mann–Whitney U test was applied to compare the values of skin surface temperature, vein diameter and changes in values of skin surface temperature (ΔT_avg_) and vein diameter (ΔD_avg_) before and after HILT. Correlations between differences in values of skin surface temperature and vein diameter after HILT application in the two groups were calculated using the Spearman’s rank correlation coefficient, rho. The results were considered significant when *p* < 0.05.

## 3. Results

### Skin Surface Temperature and Vein Diameter

All the horses completed the HILT treatment and all measurements. No statistically significant differences were found between group A and group B in the skin surface temperature of the lateral aspect of fetlock joint and diameter of the lateral palmar digital vein before HILT application.

After HILT application, there were no statistically significant differences in the skin surface temperature and vein diameter between group A and group B (*p* = 0.496 and *p* = 0.596, respectively; Table 1).

**Figure 3:**
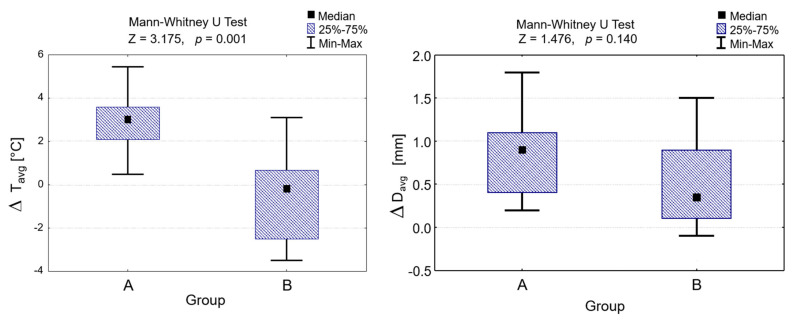
Differences in values of skin surface temperature and vein diameter after HILT application in group A and group B and the Mann–Whitney U test results. ΔT_avg_, differences in values of skin surface temperature after HILT; ΔD_avg_, differences in values of vein diameter after HILT.

**Figure 4:**
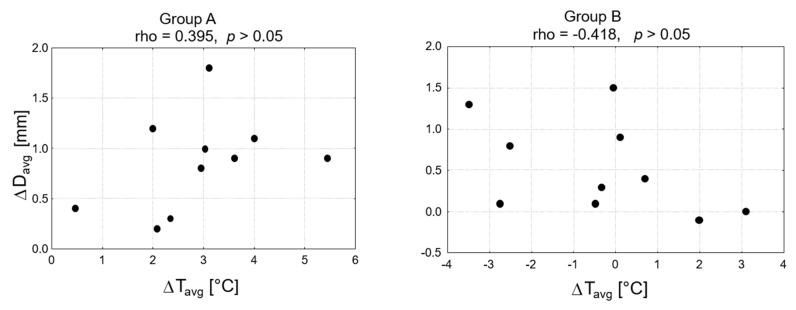
Correlation between the differences in values of skin surface temperature and vein diameter after HILT application in group A and group B, and results of Spearman’s rank correlation coefficient. ΔT_avg_, differences in values of skin surface temperature after HILT; ΔD_avg_, differences in values of vein diameter after HILT.

The comparison between group A and group B in the skin surface temperature changes and vein diameter changes before and after HILT shows that the changes in skin surface temperature were statistically significant (Z = 3.175, *p* < 0.001; Table 1, Figure 3), but the changes in vein diameter were not (Z = 1.476, *p* = 0.140; Table 1, Figure 4). However, the changes in values of the vein diameter before and after HILT were higher in group A than those in group B, with medians of 0.9 mm (IQR 0.4–1.1 mm) and0.4 mm (IQR 0.1–0.9 mm), respectively.

There was no correlation between changes in the average skin surface temperature and vein diameter in both groups (rho = 0.395 for group A and r = -0.418 for group B, *p* > 0.005; Figure 4).

## 4. Discussion

To the best of our knowledge, this is the first clinical study that documents a difference in the effects of HILT performed on pigmented and non-pigmented skin in the treatment area in clinically healthy racehorses. In our study, we found an increase in skin surface temperature in horses with pigmented skin and a decrease in horses with non-pigmented skin. The vein diameter increased in group A and group B after HILT, but the difference between both groups was not statistically significant. Finally, we did not find a correlation between the changes in skin surface temperature and vein diameter in both groups of horses.

The reaction of the skin to laser radiation depends on many physical and chemical factors [32]. Among them are skin color and the amount of melanin located in the epidermis. Melanin absorbs the visible and near-infrared part of the optical spectrum, and the absorbed energy can be transformed into heat, resulting in photothermal effects [11]. In our previous study on racing Thoroughbreds, we found a significant increase in temperature of the dorsal skin surface overlying the tarsal joint after HILT application (the mean temperature change was 2.5 °C), but the study did not control for skin pigmentation in the treatment area [28]. Additionally, in our pilot study on the use of thermography for the assessment of HILT in racehorses, we indicated a statistically significant increase in the average surface temperature of the fetlock joint (by an average of 3.0 °C) after HILT treatment [33]. Similar results were described by Bergh et al. [34] in their research on the influence of defocused CO_2_ laser therapy on the temperature in skin, subcutaneous tissue and the fetlock joint. The experiment was performed on Standardbred trotters and designed as a cross-over study with randomized laser and control treatments (with no laser output). There were significantly higher skin and subcutis temperatures in the laser-treated group, but with no significant differences between the groups in the fetlock joint temperature. In the laser-treated group, the average increase in skin temperature on the dorsal side of the fetlock joint measured during treatment was 5.3 °C ± 1.4 °C (with no information provided about skin pigmentation in the treatment area). In our study, changes in the temperature of the skin surface were lower: median 3.0 °C (IQR 2.1–3.6 °C) and median −0.2 °C (IQR −2.5–0.7 °C) for the pigmented and non-pigmented skin groups, respectively. We used much lower HILT power parameters than those used by Bergh et al. [34] with a similar time of irradiation (25 J/cm^2^ and 4 W compared to 91 J/cm^2^ and 16 W).

The decrease in skin surface temperature in the non-pigmented skin group was probably caused by the skin cooling during the experiment after both the shaving of the treatment area and use of coupling ultrasound gel. The cooling effect likely took place in both groups and continued for longer than the time it took us to perform HILT therapy and all measurements. The HILT treatment increased the skin surface temperature by a greater amount in the pigmented skin group than in the non-pigmented skin group. The limited absorption of laser radiation by white skin did not allow for the complete inhibition of the cooling effect. In our opinion, the lack of a control group that receives no HILT treatment is a major limitation of our study. It would be beneficial to have a better control of skin surface temperature changes caused by shaving and ultrasound examination.

When a treatment program is designed with a thermal modality, it is important to predict the risk of thermal exposure to the tissue. Increasing the wavelength of the laser light decreases the absorption by melanin; therefore, the near-infrared wavelength range is best suited to non-invasive treatment [35]. In our current study, we used an 808 nm wavelength and a high energy density of 25 J/cm^2^. None of the horses from either group showed discomfort or pain behavior during therapy, experienced skin burns or developed swelling in the treatment area. Therefore, HILT performed on healthy tissue was classified as a safe procedure, which was well tolerated by the horses. However, the present study lacks long-term follow-up data, which reduces the clinical application of our findings to the assessment of the short-term effects of HILT.

The vein diameter findings corroborate the results of our previous study (the mean vein diameter change was 0.75 mm) [28]. In the present study, the vein diameter also increased in group A and group B by medians of 0.9 mm (IQR 0.4–1.1 mm) and 0.4 mm (IQR 0.1–0.9 mm), respectively, but the differences between both groups were not significant. The measurement of changes in blood vessel diameter can assess the degree of vasodilatation and the increase in blood flow in tissues [36]. It is known that thermal effects enhance the tissue blood supply [12,13,14]. It is possible that during the HILT treatment in horses from the pigmented skin group, warmth receptors were stimulated, which may be important in the axon reflex vasodilatation response [37,38]. An increase in vein diameter with a simultaneous decrease in skin surface temperature in horses from non-pigmented skin group points to the fact that the thermal effect of HILT treatment is not the only contributor to an increase in blood flow [5]. It has been suggested that the laser light acts immediately on the sympathetic nervous system, causing vasoconstriction [39], but this is followed by a sympathetic-controlled reaction, resulting in vasodilation. Another explanation for this phenomenon could be an increase in production of local nitric oxide (NO). NO signalling plays an important role in the light-induced changes in the vascular diameter. Samoilova et al. [23] demonstrated that exposing human skin to a visible spectrum of light resulted in an increased microcirculation via a NO synthetase-dependent mechanism.

This study indicated no correlation between the changes in skin surface temperature and vein diameter in both groups of horses. Our earlier study demonstrated a correlation between these two parameters [28]. The lack of correlation in the non-pigmented skin group is associated with the unexpected decrease in skin surface temperature after HILT application. A possible explanation of no correlation between both parameters in horses from the pigmented skin group is the laser wavelength used. In our previous study, we used both 808 nm and 980 nm wavelengths, which were delivered simultaneously [28]. For the 808 nm wavelength, a similar energy dose and power output were used in both studies. In our previous study, additional light energy was delivered at a 980 nm wavelength with a 15 J/cm^2^ energy density and 4W of power output. A dark skin colour significantly attenuates the incident laser light that reaches the deeper skin tissue [16], and a longer wavelength penetrates deeper into the skin [40,41,42]. Macedo et al. [43] illustrated this principle in their study, which indicated that epidermal melanin absorbs approximately four times as much energy when irradiated by a 694 nm wavelength laser light than when exposed to the 1064 nm laser beam, thus allowing for a greater penetration of the longer wavelength into the dermis. In our study, a shorter wavelength (808 nm) was used (compared to both 808 nm and 980 nm wavelengths used in our previous study [28]) and was more strongly retained in melanosomes (causing intensive skin surface heat up); in addition, it caused a reduction in the amount of laser energy delivered to the deeper tissues in order to induce vasodilation strong enough to obtain a positive correlation between these two parameters.

The interquartile range of the skin surface temperature and vein diameter after HILT application in both groups of horses is large. This fact may indicate that the tissue reaction on HILT partly depends on melanin light absorption and its content in the skin. Additionally, there can be differences in melanin content within the pigmented and non-pigmented assessment. Notwithstanding, other photochemical mechanisms may also have an effect on the skin surface and vein diameter changes occurring in irradiated tissue. However, it should be kept in mind that skin pigmentation is an easily identifiable factor for the clinician and that our observations may be relevant when considering proper and safe HILT parameters for individual patients.

Limitations of the present study include a lack of sham group receiving no HILT treatment. We did not directly monitor the distal limb blood flow, although we showed vein diameter responses for HILT treatment. This study assessed short-term effects of HILT performed on healthy tissue. It would be beneficial to measure the skin surface temperature and vein diameter changes for a longer time after HILT treatment. Furthermore, we tested only healthy horse tissue as opposed to that of patients. Future research should investigate the long-term effects and benefits of HILT in horses with musculoskeletal diseases and injuries.

## 5. Conclusions

In our study, we found a significant increase in skin surface temperature in horses with pigmented skin compared to horses with non-pigmented skin. This suggests that the melanin content in the epidermis plays an important role in light energy absorption, and that properly designed laser treatments with appropriate parameters are necessary for effective and safe therapy. Further research is therefore necessary in order to describe the physiological and clinical effects of HILT performed on pigmented and non-pigmented skin.

## Figures and Tables

**Table 1:** Skin surface temperature, vein diameter and differences in values of skin surface temperature and vein diameter (median and IQR) before and after HILT application for horses from pigmented skin group (A) and non-pigmented skin group (B).

Parameters	Group A Pigmented Skin	Group B Non-Pigmented Skin	*p*-Value
T_avg_ before HILT (°C)	25.8 (23.0–29.5)	30.1 (22.1–31.8)	0.257
Min–Max	21.3–30.8	17.8–32.2	
D_avg_ before HILT (mm)	3.1 (2.8–3.7)	3.8 (2.9–4.3)	0.226
Min–Max	2.4–4.1	2.6–5.7	
T_avg_ after HILT (°C)	28.2 (26.1–31.6)	27.8 (22.9–30.7)	0.496
Min–Max	24.3–33.9	20.9–31.9	
D_avg_ after HILT (mm)	4.0 (3.5–4.9)	4.3 (3.2–5.5)	0.596
Min–Max	2.7–5.0	2.5–5.8	
ΔT_avg_ (°C)	3.0 (2.1–3.6)	−0.2 (–2.5–0.7)	0.001 *
Min–Max	0.5–5.5	–3.5–3.1	
ΔD_avg_ (mm)	0.9 (0.4–1.1)	0.4 (0.1–0.9)	0.140
Min–Max	0.2–1.8	−0.1–1.5	

* Statistically significant results of the independent non-parametric Mann–Whitney U test. Abbreviations: HILT, high-intensity laser therapy; T_avg_, skin surface temperature; D_avg_, vein diameter; ΔT_avg_, differences in values of skin surface temperature after HILT; ΔD_avg_, differences in values of vein diameter after HILT.

## Data Availability

Data is contained within that article.
